# MYC-related microRNAs signatures in non-Hodgkin B-cell lymphomas and their relationships with core cellular pathways

**DOI:** 10.18632/oncotarget.25707

**Published:** 2018-07-03

**Authors:** Giorgio Malpeli, Stefano Barbi, Gabriele Tosadori, Corinna Greco, Simonetta Zupo, Serena Pedron, Matteo Brunelli, Anna Bertolaso, Maria Teresa Scupoli, Mauro Krampera, Paul Takam Kamga, Carlo Maria Croce, George Adrian Calin, Aldo Scarpa, Alberto Zamò

**Affiliations:** ^1^ Department of Surgical Sciences, Dentistry, Gynecology and Pediatrics, Section of Surgery, University of Verona, Verona, Italy; ^2^ Department of Diagnostics and Public Health, University of Verona, Verona, Italy; ^3^ Center for BioMedical Computing, University of Verona, Verona, Italy; ^4^ Department of Medicine, Section of Hematology, Stem Cell Research Laboratory, University of Verona, Verona, Italy; ^5^ Laboratory of Molecular Diagnostics, IRCCS-AOU San Martino-IST, Istituto Nazionale per la Ricerca sul Cancro, Genoa, Italy; ^6^ Department of Medicine, Section of Hematology, University of Verona, Verona, Italy; ^7^ Department of Molecular Virology, Immunology and Medical Genetics, Comprehensive Cancer Center, The Ohio State University, Columbus, OH, USA; ^8^ Department of Experimental Therapeutics and The Center for RNA Interference and Non-Coding RNAs, The University of Texas MD Anderson Cancer Center, Houston, TX, USA; ^9^ Applied Research on Cancer-Network (ARC-NET), University of Verona, Verona, Italy; ^10^ Department of Oncology, University of Turin, Torino, Italy

**Keywords:** microRNAs, MYC, non-Hodgkin B-cell lymphoma, cell lines, network analysis

## Abstract

In order to investigate the role of microRNAs in the pathogenesis of different B-cell lymhoma subtypes, we have applied an array-based assay to a series of 76 mixed non-Hodgkin B-cell lymphomas, including Burkitt’s lymphoma (BL), diffuse large B-cell lymphoma, primary mediastinal B-cell lymphoma, mantle cell lymphoma (MCL) and follicular lymphoma. Lymphomas clustered according to histological subtypes, driven by two miRNA clusters (the miR-29 family and the miR-17-92 cluster). Since the two miRNA clusters are known to be MYC-regulated, we investigated whether this would be supported in MYC-driven experimental models, and found that this signature separated BL cell lines and a *MYC*-translocated MCL cell lines from normal germinal center B-cells and other B-cell populations. Similar results were also reproduced in tissue samples comparing BL and reactive lymph node samples. The same series was then quantitatively analyzed for MYC expression by immunohistochemistry and MYC protein levels were compared with corresponding miRNA signatures. A specific metric was developed to summarize the levels of MYC-related microRNAs and the corresponding protein levels. We found that MYC-related signatures are directly related to MYC protein expression across the whole spectrum of B-cells and B-cell lymphoma, suggesting that the MYC-responsive machinery shows predominantly quantitative, rather than qualitative, modifications in B-cell lymphoma. Novel MYC-related miRNAs were also discovered by this approach. Finally, network analysis found that in BL MYC-related differentially expressed miRNAs could control, either positively or negatively, a limited number of hub proteins, including BCL2, CDK6, MYB, ZEB1, CTNNB1, BAX and XBP1.

## INTRODUCTION

Non-Hodgkin’s B-cell lymphomas (NHBCL) arise following clonal expansion and consequent invasion of immune organs by B-cells blocked at a certain step of the differentiation process. Genetic abnormalities support the transformed state of B-cells in parallel with changes in gene expression [1]. Gene expression alterations were identified in many NHBCL types, although the mechanisms of transcription regulation sustaining the transformed state are of difficult comprehension [2, 3].

MicroRNAs (miRNAs) are a class of short noncoding single-stranded RNA implicated in the regulation of mRNA synthesis and translation. Each miRNA can bind and regulate multiple transcripts and a transcript is under potential control by multiple miRNAs [4]. MiRNAs are typical pleiotropic agents which together with other transcriptional regulators and the epigenetic machinery, are critical for setting the levels of proteins for proper cellular functions [5]. The net outcome of all actions entails a network of regulatory circuitries, including proteins and RNAs fine tuning cellular functions for the adaptation to different requirements.

MiRNAs are important for the regulation of translation in physiological and pathological states, including the sequential differentiation of B-cells and lymphomagenesis [6, 7]. Multiple miRNAs, including the miRNA *miR-17-92* cluster, *miR-34a*, *miR-125b*, *miR-150*, *miR-181a*, and *miR-212/132*, are essential for a correct early B-cell development [8]. During late B-cell maturation, downregulation of *miR-150* is required for germinal center (GC) selection and development of the adaptive humoral immune response [9]. Other miRNAs as *miR-155*, *miR-181b*, *miR-15a*, *miR-28*, *miR-16*, *miR-15b*, *miR-34a*, *miR-9*, *miR-30*, *let-7a*, *miR-125b*, *miR-217* and *miR-185*, modulate the expression of key proteins essential for the B-cell maturation [7].

The oncogene MYC can bind 10–15% of the genetic loci including many miRNAs [10]. MYC is a double face transcription factor since on one hand it supports cell proliferation and on the other apoptosis. Aberrant function of MYC is a feature of about 60% of all human cancers; *MYC* translocations are present in most Burkitt’s lymphomas [11–13] and in 5–15% of DLBCL [14]; *MYC* gains or amplification and rarely *MYC* translocation were complexively detected in 36% of MCL [15]. *MYC* mutations were found in 70% of BL and in 16% of DLBCL [16, 17]. The combined effect of *MYC* translocation and specific mutations associate with variable clinical outcome in DLBCL [17].

MYC is a potent modulator of transcription of miRNAs and *vice versa* [18]. The identification and role of MYC-regulated miRNAs was performed in MYC-inducible cell lines models of B-cell lymphoma [19, 20]. Histone deacetylation is involved in MYC mediated transcriptional repression. MYC, HDAC3, and PRC2 were demonstrated to form a repressive complex tethered to *miR-29* and *miR-26a* promoter elements to epigenetically repress transcription of these miRNAs in MYC-expressing lymphoma cells [21]. Enforced expression of repressed miRNAs diminished the tumorigenic potential of lymphoma cells indicating that MYC-repressed miRNAs function as tumor suppressor genes. Among miRNAs regulated by MYC, the *miR-17-92* cluster has oncogenic effects dependent from its ability to stimulate the cell cycle progression. Precise doses of MYC are able to stimulate cell proliferation instead of apoptosis [22]. MYC stimulates the BCR response via the upregulation of *miR-17-92* cluster and subsequent suppression of inhibitors required to limit BCR. This BCR stimulation resulted in a lymphomagenic feed-forward regulatory loop [23]. The miRNA signature associated to *MYC* has been characterized in cellular models [19], in liver cancer [24], in neuroblastoma [25], in lymphomas known to overexpress MYC such as Burkitt’s lymphoma and diffuse large B-cell lymphomas [26] and by computational methods [27]. These studies applied different approaches to reveal the MYC-miRNA connection and focused on specific aspects.

MiRNAs take part in regulatory networks affecting proteins level and cellular processes. To contribute to clarify the implication of miRNAs in malignant B-cell transformation, we first compared the miRNA profiles of Burkitt’s lymphoma (BL), diffuse large B-cell lymphoma (DLBCL), primary mediastinal B-cell lymphoma (PMBL), mantle cell lymphoma (MCL) and follicular lymphoma (FL). We identified miRNA signatures able to discriminate NHBCLs that involved known MYC targets. To assess if this miRNA signature was independent from the specific microenvironment of NHBCLs, six BL and two MCL cell lines were compared with normal B-cells as reference and BL tissues were compared with reactive lymph nodes. To study known and new *MYC* signatures connected with miRNAs profile of NHBCLs, we investigated MYC expression by immunohistochemistry (IHC) and correlated the results with miRNAs levels. Finally, we performed network analysis to track down the protein-miRNAs network modulated by differentially expressed miRNAs in NHBCLs.

## RESULTS

### Differences of miRNA signatures in non-Hodgkin’s B-cell Lymphoma types

**Figure 1:**
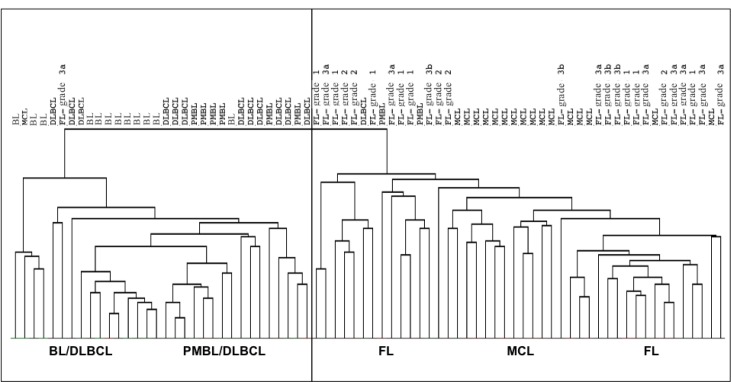
Distribution of 76 samples belonging to BL, DLBCL, PMBL, MCL and FL according to their miRNA profile

**Figure 2:**
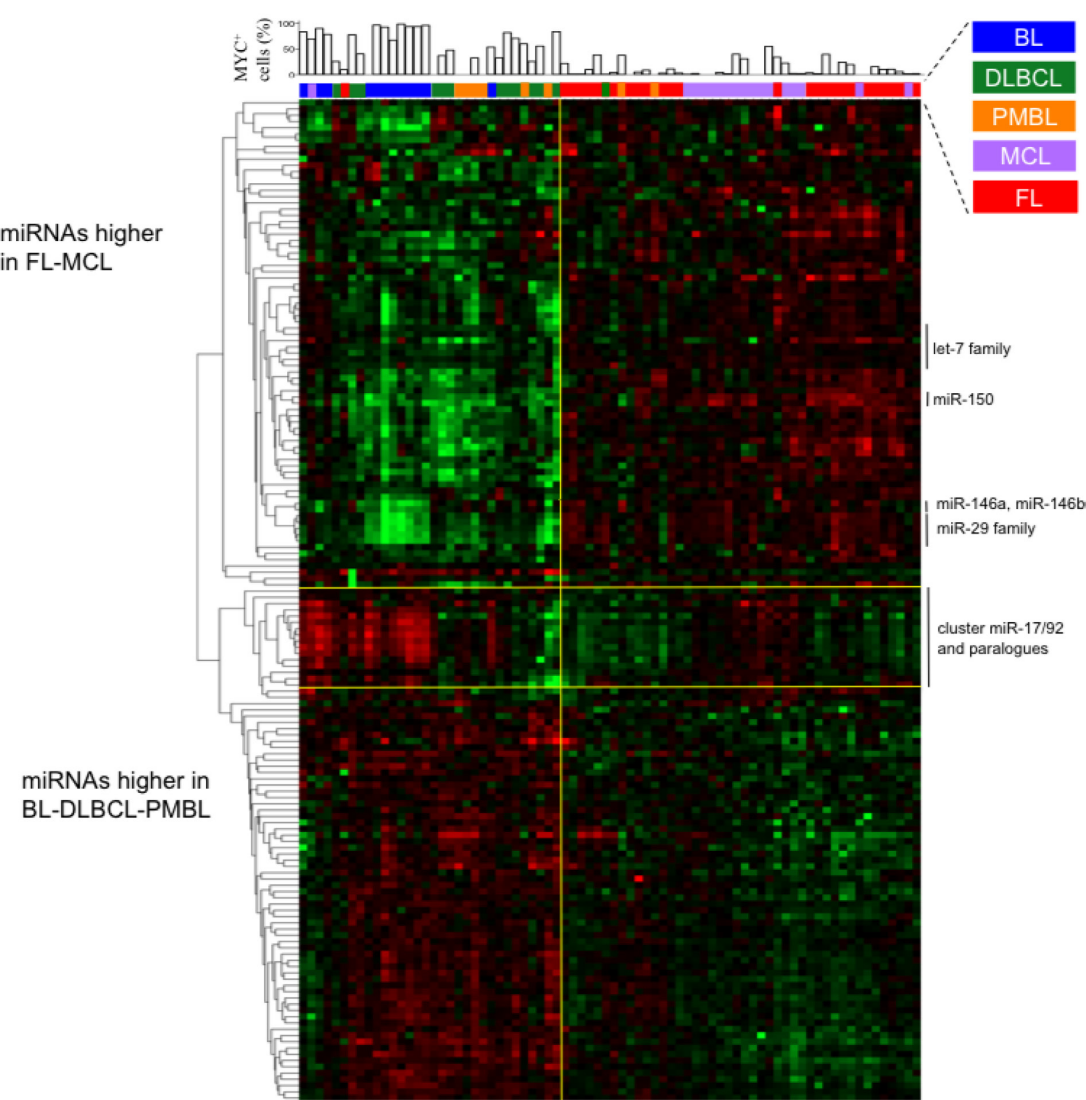
Levels of miRNAs differentially expressed among BL, DLBCL, PMBL, MCL and FL samples The heat map describes the expression levels of 110 single miRNAs differentially expressed among five lymphoma types at FDR 0.5%. At the top of the heat map, for each sample is indicated the per cent of MYC^+^ cells detected by immunohistochemistry.

We investigated the miRNAs profile in different NHBCLs types having origin from follicular *naive* or germinal center (GC) B-cells. We compared 76 NHBCL samples comprising 12 Burkitt’s lymphoma (BL), 13 diffuse large B-cell lymphoma (DLBCL), 8 primary mediastinal B-cell lymphoma (PMBL), 17 mantle cell lymphoma (MCL) and 26 follicular lymphomas (FL) (Figures 1 and 2). According to the miRNA profiles, intratype heterogeneity was shown in each NHBCL type. Clusterization procedures split samples in two large clusters: a cluster included mainly BL, DLBCL and PMBL; the other cluster included mainly FL and MCL cases. A total of 110 miRNAs subdivided in three clusters were differentially expressed among the five NHBCL types at FDR 0.5%, fold change >1.5, (Figure 2). One miRNA cluster included miRNAs upregulated in MCL and FL. A second cluster included miRNAs upregulated in BL, DLBCL and PMBL. A third miRNA cluster encompassed mainly miRNAs of the *miR-17-92* cluster and paralogues. These miRNAs were expressed at a higher level in BL and in a minor portion of DLBCL, PMBL, MCL and FL cases. The polycistron *miR-17-92* cluster, *miR-29* family, *miR-150* and *miR-497* showed the highest power of discrimination of the five NHBCL types (Table 1).

**Table 1:** MiRNA differentially expressed among BL, DLBCL, PMBL, FL and MCL

miRNA	*Q* value	miRNA	*Q* value
*miR-17-5p*	4.9E-14	*mir-133b*	1.8E-05
*miR-29c*	1.3E-13	*miR-125b*	1.8E-05
*miR-106a*	1.4E-13	*mir-184*	1.9E-05
*miR-20b*	3.2E-13	*miR-338*	1.9E-05
*miR-106b*	5.0E-13	*miR-370*	2.1E-05
*let-7a*	1.2E-12	*mir-335*	3.1E-05
*miR-150*	1.4E-12	*mir-431*	3.5E-05
*miR-497*	1.4E-12	*miR-15a*	4.2E-05
*miR-20a*	1.5E-12	*miR-126*	4.9E-05
*miR-29a*	1.7E-12	*miR-200c*	5.2E-05
*miR-29b*	1.0E-11	*let-7g*	5.6E-05
*miR-93*	1.7E-11	*miR-192*	6.1E-05
*miR-342*	2.2E-11	*mir-331*	7.1E-05
*miR-27a*	2.3E-11	*miR-30e-5p*	8.5E-05
*miR-374*	4.0E-11	*miR-32*	8.5E-05
*miR-26a*	6.7E-11	*miR-218*	1.0E-04
*mir-339*	1.2E-10	*miR-498*	1.1E-04
*miR-26b*	2.3E-10	*miR-30c*	1.1E-04
*miR-146b*	3.3E-10	*miR-24*	1.2E-04
*mir-206*	4.5E-10	*miR-135a*	1.9E-04
*miR-146a*	5.0E-10	*miR-99b*	2.0E-04
*miR-194*	5.6E-10	*miR-224*	2.0E-04
*miR-221*	1.1E-09	*mir-34c*	2.0E-04
*mir-196b*	1.2E-09	*miR-410*	2.2E-04
*miR-326*	1.5E-09	*miR-199a*	2.3E-04
*miR-18a*	1.5E-09	*miR-99a*	2.4E-04
*miR-7*	3.0E-09	*mir-424*	2.6E-04
*miR-328*	6.1E-09	*miR-28*	3.3E-04
*miR-212*	7.0E-09	*miR-409-3p*	3.4E-04
*mir-140*	8.2E-09	*miR-30b*	3.6E-04
*miR-361*	1.2E-08	*miR-196a*	4.5E-04
*let-7d*	2.2E-08	*miR-98*	5.4E-04
*miR-19b*	2.4E-08	*mir-193a*	5.6E-04
*miR-30d*	3.9E-08	*miR-181d*	5.6E-04
*mir-10b*	6.9E-08	*mir-204*	5.6E-04
*let-7f*	7.9E-08	*miR-485-3p*	6.1E-04
*miR-128a*	9.7E-08	*miR-215*	6.2E-04
*miR-219*	1.0E-07	miR-9^*^	6.3E-04
*mir-340*	1.3E-07	*miR-181c*	6.5E-04
*miR-346*	1.3E-07	*miR-143*	6.5E-04
*miR-499*	1.3E-07	miR-126^*^	6.6E-04
*miR-21*	1.3E-07	miR-373^*^	6.7E-04
*miR-9*	1.3E-07	*miR-153*	6.8E-04
miR-202^*^	1.5E-07	*miR-15b*	8.3E-04
*miR-125a*	1.6E-07	*mir-96*	8.5E-04
*miR-10a*	2.2E-07	*miR-527*	9.3E-04
*mir-152*	4.1E-07	*miR-101*	9.3E-04
*miR-30a-3p*	4.1E-07	*miR-181a*	1.1E-03
*let-7c*	4.2E-07	*miR-301*	1.1E-03
*miR-142-5p*	6.1E-07	miR-181a^*^	1.3E-03
*miR-155*	6.7E-07	*miR-181b*	1.4E-03
*mir-320*	8.6E-07	*miR-494*	1.4E-03
*miR-483*	1.1E-06	*mir-132*	1.4E-03
*mir-422a*	1.3E-06	*miR-195*	1.5E-03
*miR-92*	2.1E-06	*mir-345*	1.5E-03
*miR-222*	2.7E-06	*miR-429*	1.5E-03
*mir-185*	3.3E-06	*miR-17-3p*	1.5E-03
*mir-130b*	5.3E-06	*miR-324-3p*	1.9E-03
*miR-30a-5p*	6.0E-06	*miR-214*	1.9E-03
*miR-100*	6.1E-06	*miR-16*	2.0E-03
*miR-25*	6.3E-06	*miR-136*	2.1E-03
*miR-330*	6.7E-06	*miR-500*	2.2E-03
*miR-324-5p*	7.7E-06	*mir-496*	2.3E-03
*miR-129*	8.2E-06	*miR-191*	2.4E-03
*miR-425-3p*	1.3E-05	*miR-147*	2.7E-03
*miR-23a*	1.4E-05	*miR-27b*	2.7E-03
*mir-211*	1.6E-05	*miR-23b*	3.1E-03
*miR-210*	1.7E-05	*miR-122a*	3.9E-03

### Strong up-regulation of *miR-17-92* cluster and downregulation of *miR-221*, *miR-222*, *miR-223* and *miR-224* in BL and MCL cell lines compared to normal B-cells

**Figure 3:**
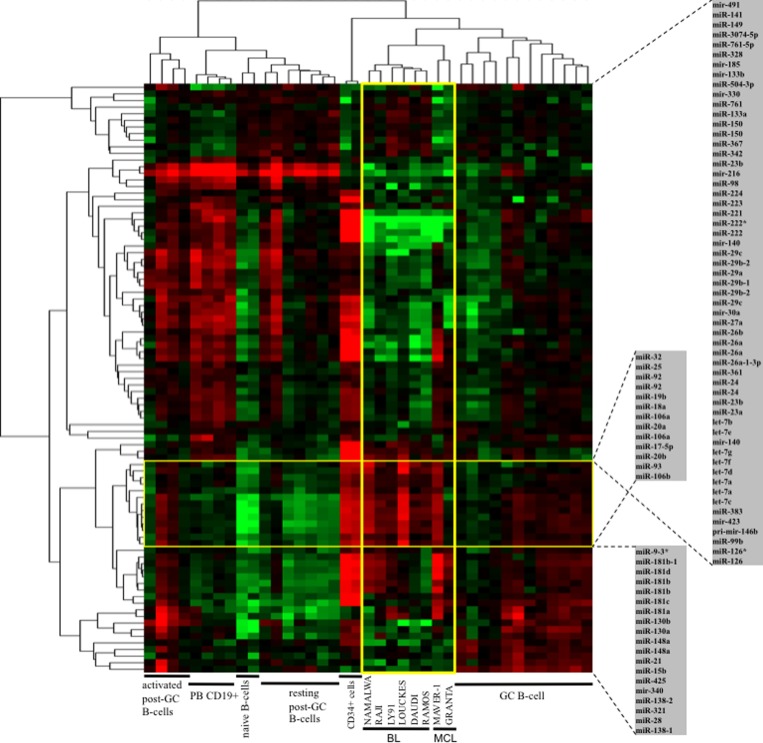
MiRNAs expression profiles of BL and MCL cell lines compared to bone marrow CD34^+^ cells and normal B-cells at different stages of differentiation MiRNAs expression profile of six BL cell lines and two MCL cell lines compared with that of CD34^+^ cells and normal B-cells at different differentiation stages. GC, germinal center; PB B-cells, peripheral blood B-cells.

We investigated whether the differences of miRNA profiles observed among NHBCL tissues were recapitulated in corresponding lymphoma cell lines. To capture the pathological signature in cell lines, we compared the miRNAs expression profile of six BL and two MCL cell lines (of these, one with known MYC overexpression) with normal B-cell populations at diverse differentiation stages, ranging from bone marrow CD34^+^ cells to mature post GC activated B-cells from tonsils **(**Figure 3). MiRNAs expression in normal B-cells were in part published [28].

BL and MCL cell lines clustered together and close to GC B-cells and CD34^+^ cells. Cell lines and CD34^+^ cells showed many differences but similar high levels of *miR-17-92* cluste*r*, *miR-126* and miR-126* compared to other categories. Moreover, the cell lines had lower level of *miR-221*, *miR-222*, miR-222*, *miR-223* and *miR-224* compared to other B-cell populations. *Mir-150*, which is strictly regulated during normal B-cell development, was expressed at a level that was lower in cell lines in comparison to CD34^+^ cells and GC B-cells.

BL cell lines showed homogeneous profiles: only members of the *miR-181* family, miR-9*, *miR-130a* and *miR-130b* were variably expressed. The miRNA profile of the MCL cell lines was more similar to that of BL cell lines than to that of *naive* B-cells. The main differences of miRNA expression between MCL cell lines MAVER-1 (known to overexpress MYC due to translocation) and GRANTA-519 regarded *miR-181* family and *miR-17-92* cluster. In particular, MAVER-1 but not GRANTA-519 showed levels of *miR-17-92* cluster similar to those of BL cell lines.

### MiRNA signature in Burkitt’s lymphoma tissues

**Figure 4:**
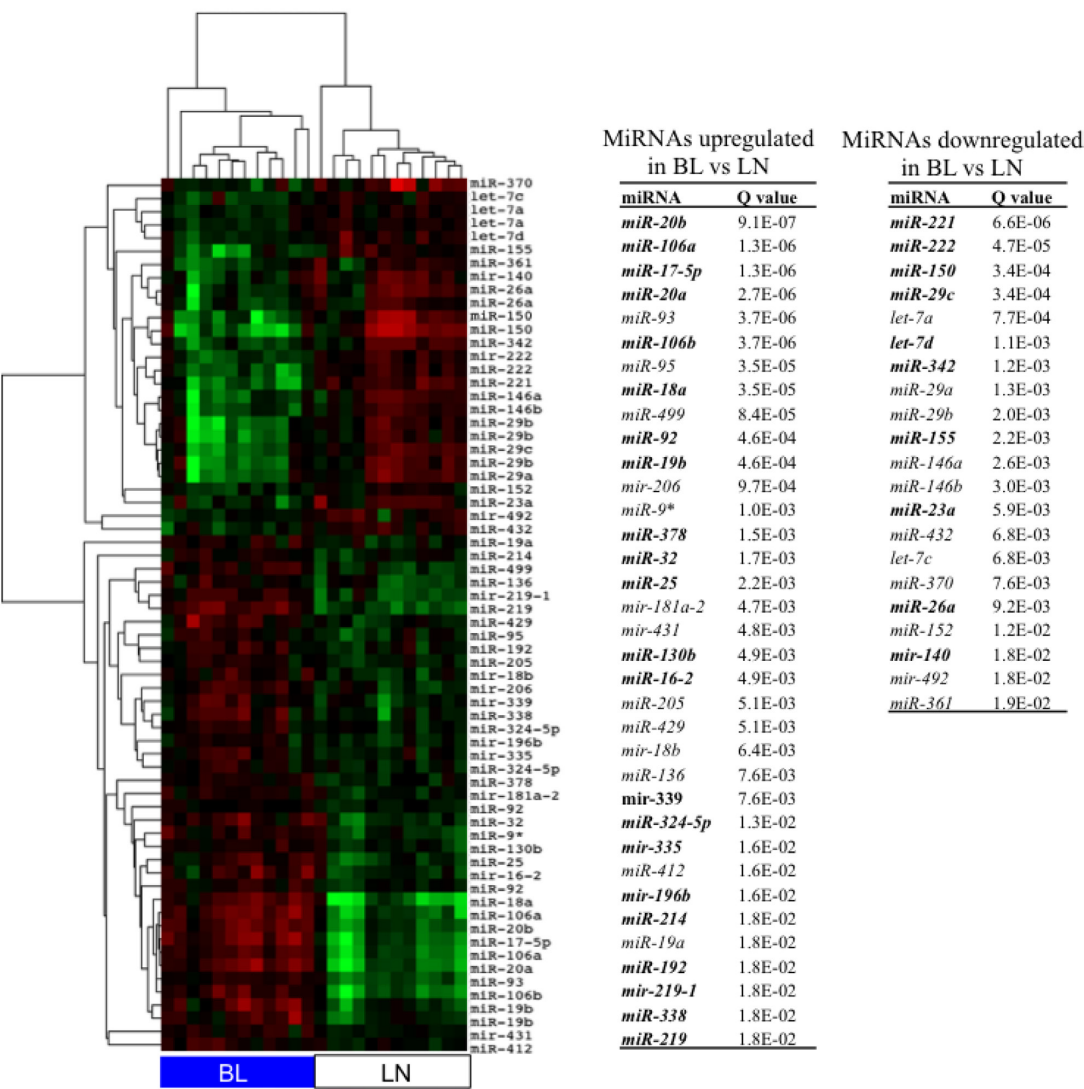
MiRNA profile of BL tissues compared to LN The heat map shows the levels of miRNAs differentially expressed between 14 BL and 11 reactive LN samples at FDR 2%. In table, the list of 56 single miRNAs differentially expressed between BLs and LNs. In bold, miRNAs differentially expressed between BL cell lines and GC B-cells.

To verify if the miRNAs signature observed in cell lines was reproduced in tissues, we compared the miRNAs expression of BL tissues and reactive lymph nodes (LNs) as normal reference. BLs clustered separately from LNs and 56 miRNAs were differentially expressed: 34 upregulated and 21 downregulated in BL at FDR 2% and fold change >1.5 (Figure 4). Top upregulated miRNAs included *mir-17-92* cluster, *miR-499*, *mir-206*, miR-9*. Top downregulated miRNAs were *miR-222*, *miR-221*, *miR-150*, *miR-29* family, *let-7* family, *miR-342*, *miR-155*, *miR-146a*, *miR-146b* and *miR-23a*.

MiRNAs deregulated in both cell lines and BL tissues were members of *miR-17-92* cluster, *miR-222*, *miR-221*, *miR-150*, *let-7* family members.

### Validation of miRNA expression in NHBCLs and LNs by quantitative RT-PCR

Expression of 9 miRNAs was validated by quantitative RT-PCR in BL, DLBCL, PMBL, MCL, FL and LN (Supplementary Figure 1). The 9 miRNAs showed significant differences in at least one NHBCL type with respect to LN (*P* < 0.05): *let-7a* in DLBCL, PMBL and BL; miR-9* in FL, MCL, DLBCL, PMBL and BL; *miR-10a* in DLBCL and PMBL BL; *miR-20b* in MCL and BL; *miR-21* in FL, MCL, DLBCL, PMBL and BL; *miR-29a* in FL, MCL and BL; *miR-150* in DLBCL, PMBL and BL: *miR-155* in FL, MCL, DLBCL, PMBL and BL; *miR-222* in FL, DLBCL, PMBL and BL.

### A *MYC* signature is deeply embedded into the miRNA profile of BL and in a fraction of other NHBCL types

**Figure 5:**
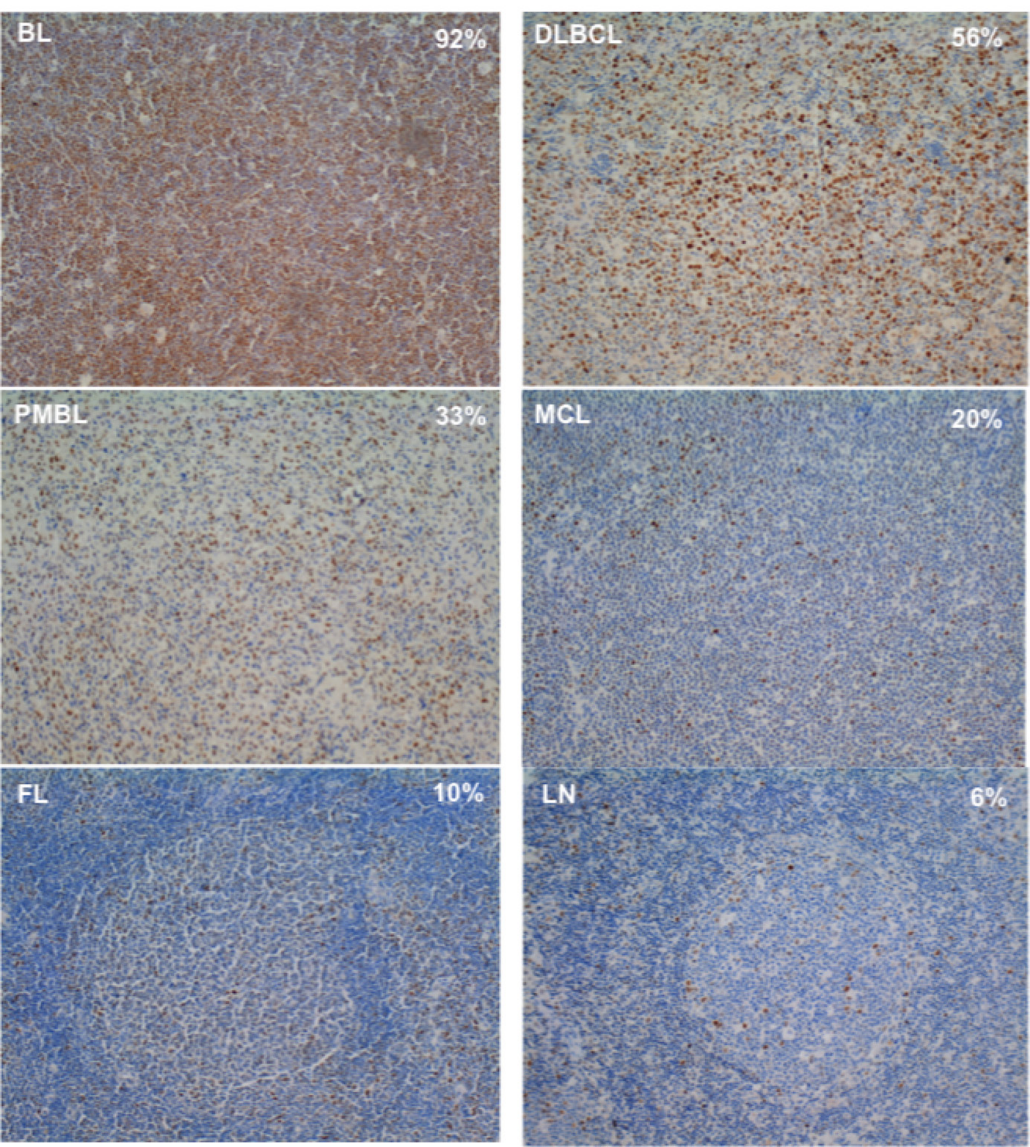
Examples of MYC stain in BL, DLBCL, PMBL, MCL, FL and LN cases MYC^*+*^ cells were quantified in 4 μm whole-tissue sections using the rabbit monoclonal antibody anti-MYC Y69 diluted 1:300. Positive cell count was performed using the cellSense Dimension software. The per cent of MYC^+^ cells having a nuclear signal above the background signal showed in each slide was the ratio between the number of MYC^+^ nuclei and 10.000 hematoxylin-stained nuclei. The background signal was assessed on slides of reactive LNs showing low frequency nuclear stain. The threshold was set just under the level that allowed to consider as negative the 95% of cells and at least 1^+^ the remaining positive cells, according to the evaluation of an expert pathologist. The per cent value reported on the picture refers to the average per cent of MYC^+^ cells of the sample.

**Figure 6:**
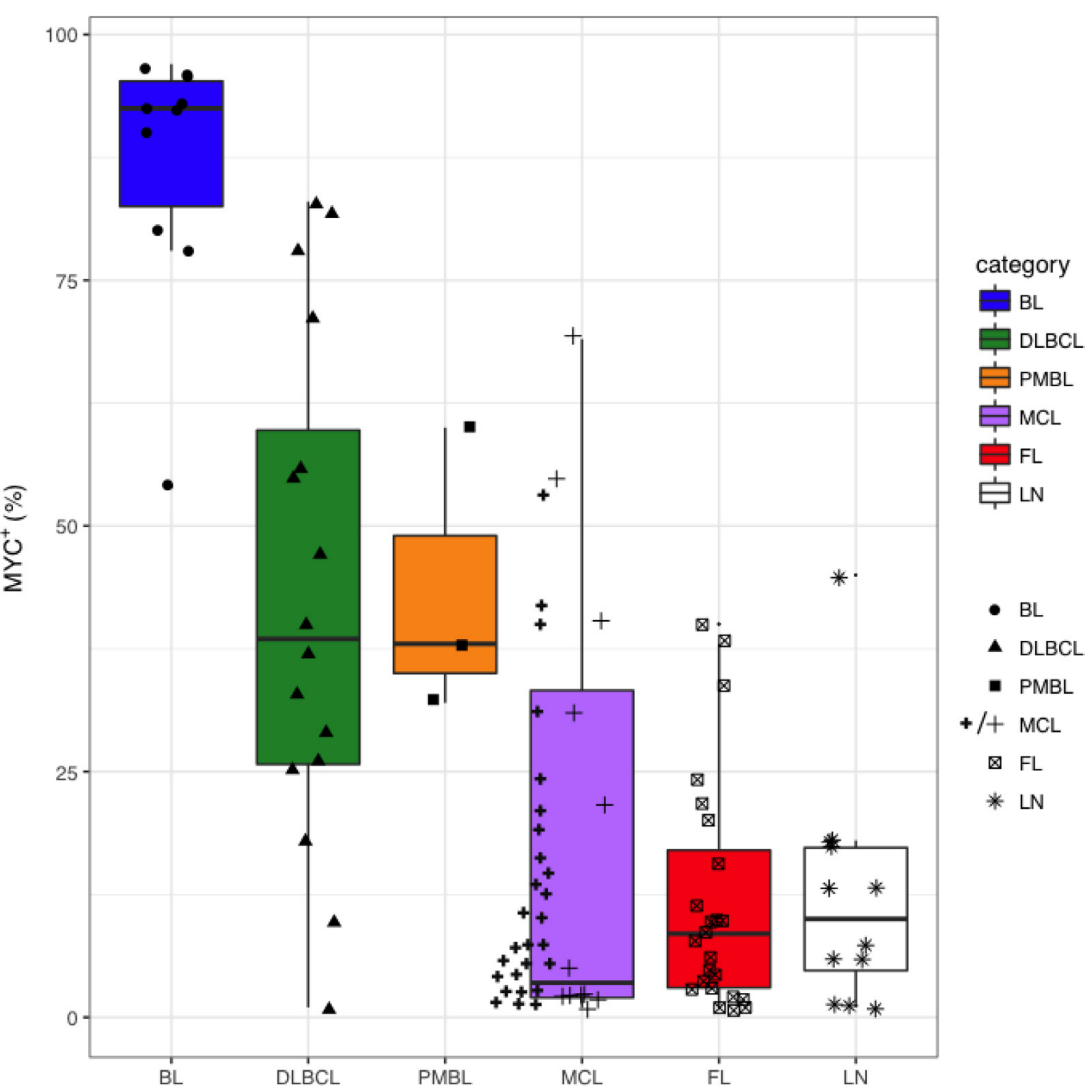
Distribution of MYC^+^ cell counts in BL, DLBCL, PMBL, MCL, FL and LN MYC expression was assessed by immunohistochemistry with anti-MYC monoclonal Ab in BL, DLBCL, PMBL, MCL, FL and LN samples and MYC^*+*^ cells were subsequently counted using the cellSense software. The per cent of MYC^+^ cells having a nuclear signal above the background signal was the ratio between the number of MYC^+^ nuclei and 10.000 hematoxylin-stained nuclei. An additional series of 27 MCL cases was assessed (thick line cross). Box and whiskers: median, 25–75% interquartile, 95% interval confidence.

**Figure 7:**
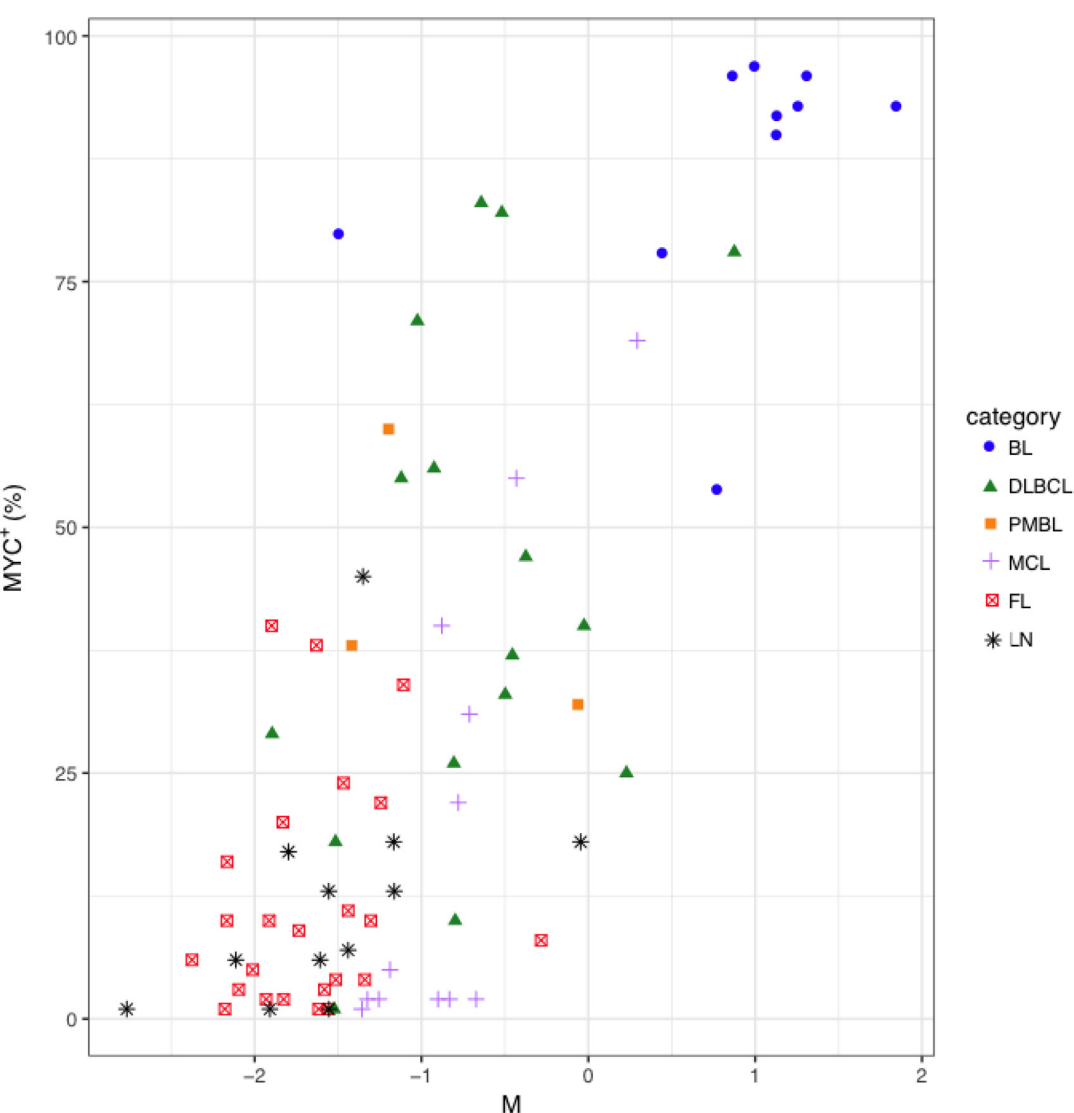
Distribution of MYC^+^ cell counts in function of the M parameter in BL, DLBCL, PMBL, MCL, FL and LN samples MYC expression was assessed by immunohistochemistry with anti-MYC monoclonal Ab in BL, DLBCL, PMBL, MCL, FL and LN and MYC^*+*^ cells were subsequently counted using the cellSense software. For each sample, *MYC*-up-regulated miRNAs and *MYC*-down-regulated miRNAs represent the average level of miRNAs known as up-repulated or down-regulated by MYC, respectively. M parameter was calculated for each sample as *MYC*-up-regulated miRNAs minus *MYC*-downregulated miRNAs.

**Figure 8:**
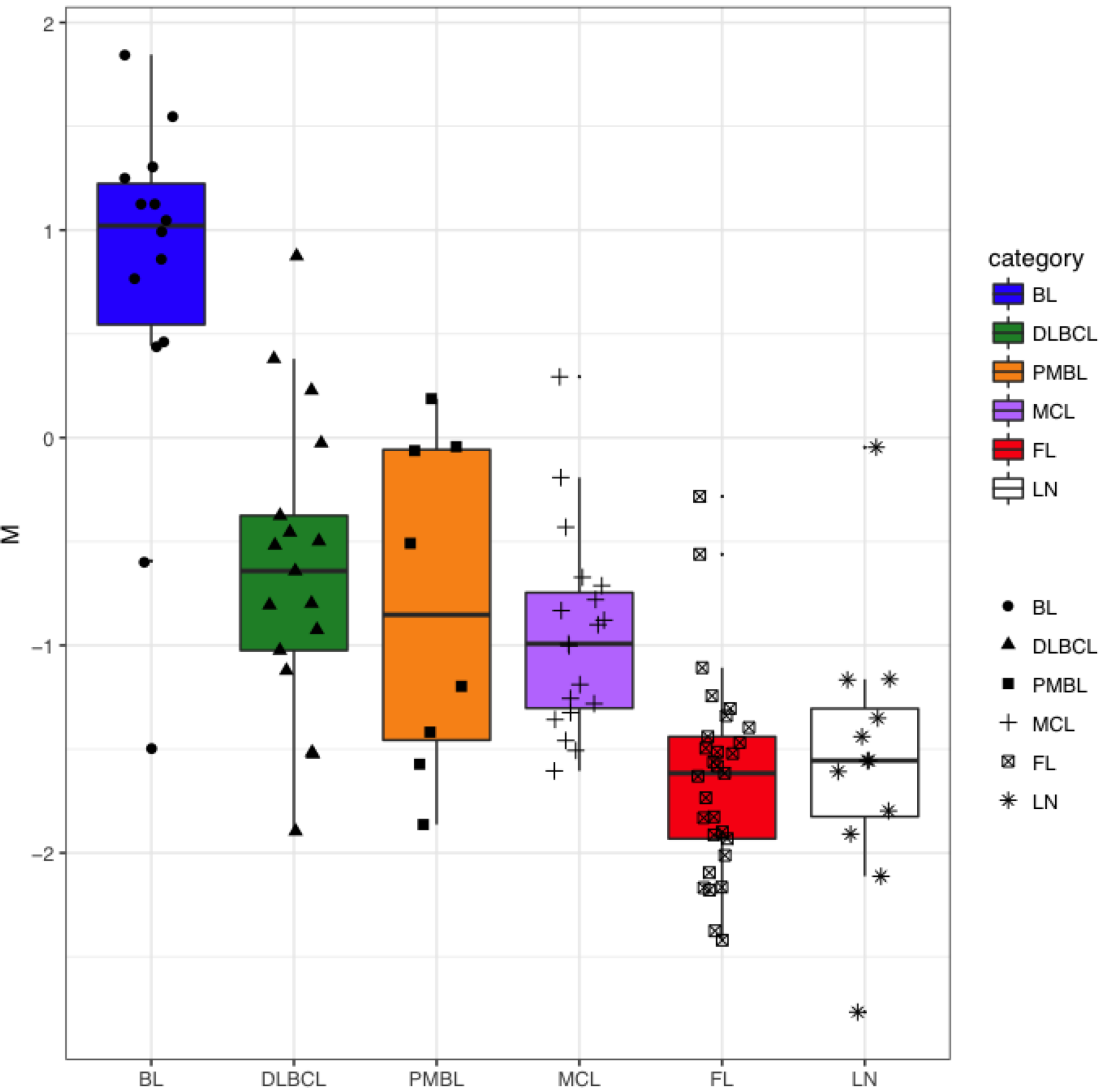
Distribution of M parameter in BL, DLBCL, PMBL, MCL, FL and LN For each sample, *MYC*-uprepulated miRNAs and *MYC*-downregulated miRNAs represent the average level of miRNAs known as uprepulated or downregulated by MYC, respectively. M parameter was calculated for each sample as *MYC*-upregulated miRNAs minus *MYC*-downregulated miRNAs.

The oncogene MYC plays a relevant pathological role in NHBCL pathogenesis [29]. In particular, BL is known as MYC-driven lymphoma. In addition, MYC was demonstrated to be ruling for miRNA expression in cellular models and lymphomas [18]. To verify if a MYC-related miRNA signature showed a phenotypic equivalent in the five types of NHBCLs under investigation, we assessed MYC expression by IHC in NHBCLs and in LN samples and correlated the MYC^+^ cell counts with the average level of miRNAs demonstrated to be under *MYC* regulation [18] (Figures 2, 5–8).

Examples of MYC stain in BL, DLBCL, PMBL, MCL and FL cases and of LN were reported in Figure 5. MYC^+^ cells rate was higher, as expected, in BL cases (median 92.5% of cells) with respect to the other NHBCL types, namely DLBCL (38.1%), PMBL (37.7%), MCL (3.8%), FL (7.9%) and LNs (9.9%). The largest variation of MYC^+^ cells was observed in DLBCL with positive cells ranging from 2% to 82%. (Figure 6).

In five of 12 (41.6%) MCL cases the fraction of MYC^+^ cells was >20%. To confirm the trend of MYC expression observed in MCL, we investigated MYC expression in 27 additional MCL cases. Here, the median of MYC^+^ fraction was 7.9% and 7 of 27 (26%) cases had >20% of MYC^+^ cells and one showed a MYC staining over 70% (Figure 6).

The transcription factor *MYC* is known to induce the downregulation of members of *let-7* family, *miR-30* family, *miR-29* family, *miR-26a* and *miR-26b*, *miR-34a*, *miR-146a*, *miR-150* and *miR-195*, and the upregulation of miRNAs of the *miR-17-92* cluster in different models [19, 20]. We wondered whether MYC^+^ cell counts were correlated with the expression level of known MYC-related miRNAs in NHBCLs and LNs. Higher MYC^+^ cell counts correlated with a higher value of what we defined the “M parameter” (MYC-upregulated miRNAs minus MYC-downregulated miRNAs) (Figure 7). This relationship was evident in all NHBCL types and among cases of the same lymphoma type. This result suggests that a large part of the heterogeneity both intra- and inter-type of miRNAs profile observed in the NHBCLs (Figure 2) can be attributed to MYC or to be its indirect consequence. As a confirmation of the reliability of parameter M, its distribution in the five types of NHBCLs was similar to that observed for MYC positivity: highest in BL, followed by DLBCL, PMBL, MCL, FL and LN, in descending order (Figure 8).

### Identification of new MYC-related miRNA expression in NHBCLs

**Figure 9:**
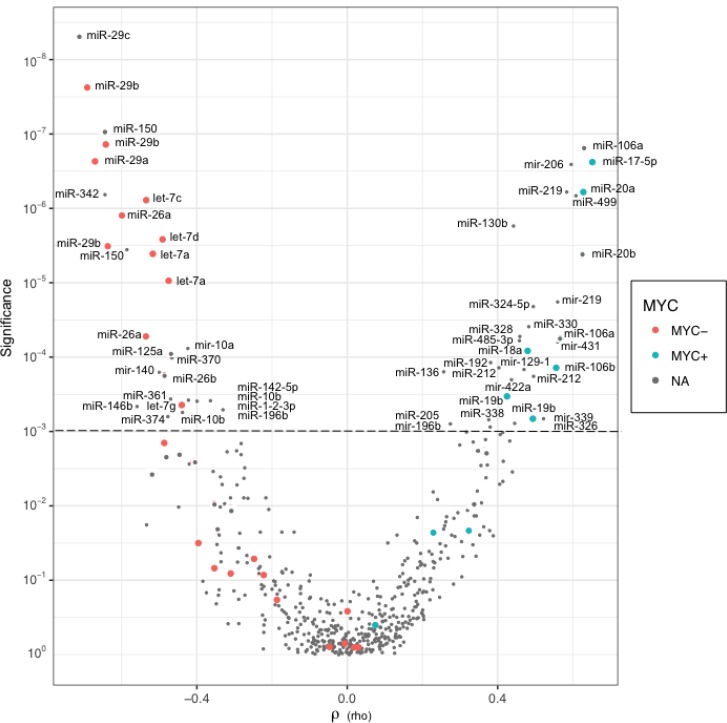
Distribution of *P* values in function of Spearman’s correlations (rho) for each miRNA probe Each point of the Volcano plot describes the linkage between the levels of a miRNA probe and the MYC^+^ cell counts in all lymphoma samples. Red points and blue points represent negative and positive correlations, respectively, of miRNAs known as *MYC*-regulated. Grey points represent correlations of miRNAs supposedly not MYC-related.

To discover new MYC-related miRNAs expressions in NHBCLs, MYC^+^ cells counts were correlated with the expression level of all miRNA probes. Distribution of P values of MYC^+^ cell counts/miRNA probe relationships in the function of Spearman’s correlation values (rho) was shown by Volcano plot (Figure 9). P values split in two arms according to positive or negative correlation. Among positive correlations, miRNAs of the *miR-17-92* cluster showed higher significance together with *mir-206*, *miR-219*, *miR-499*, *miR-130b*, *miR-328*, *miR-324-5p*, *miR-330*, *miR-485*, *mir-431*, *miR-192 and other* (*P* < 0.001). Among negative correlations, we found *miR-29* family, *miR-150*, *miR-342*, members of *let-7 family*, *miR-26a*, *miR-10a*, *miR-125a*, *miR-370, miR-140*, *miR-26b* (*P* < 0.001). A complete list of miRNAs significantly correlated with MYC^*+*^ cell counts in NHBCLs and LNs (*P* < 0.05) is reported in Supplementary Tables 1 and 2.

### *MYC* is rarely translocated in DLBCL and MCL cases with high MYC^+^ cells counts

High MYC^+^ cell counts were detected in BL, as expected, and also in a fraction of DLBCL and MCL cases (Figure 6). To assess if MYC overexpression cases could depend from a gene translocation in DLBCL and MCL, we performed fluorescence *in situ* hybridization analysis (FISH). MYC translocation was detected in 1 of 13 DLBCL (having 81% of MYC^+^ cells) and in none of the 3 MCL cases with high MYC^+^ cell counts. Instead, these 3 MCL cases showed a copy number gain of MYC (Supplementary Table 3).

### *BCL2, CDK6, ZEB1, MYB* and *BCL6* are hub proteins of cellular circuitries under control of miRNAs in NHBCLs

**Figure 10:**
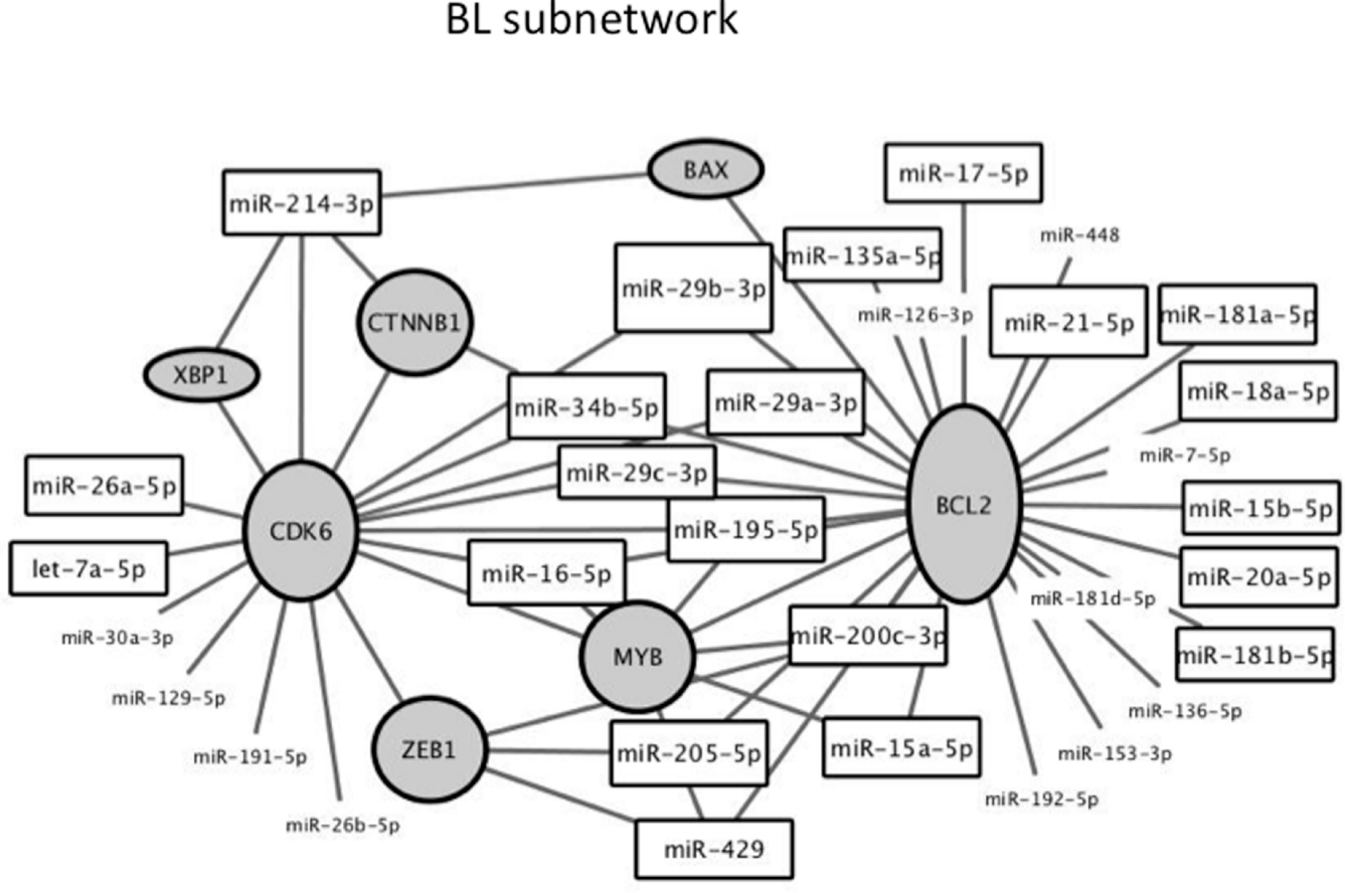
Subnetwork of proteins and miRNAs around the two hub proteins with higher degree identified by network analysis of targets of miRNA differentially expressed between BL and LN In grey are the hub proteins. Height of the frame is directly proportional to the degree. Framed miRNAs refer to miRNAs involved in the network. MiRNAs without frame refer to other miRNAs differentially expressed which targets the proteins in the subnetwork.

We performed network analysis of targets of miRNAs differentially expressed in BL. To infer proteins modulated by miRNAs, we consulted a global intracellular protein-protein interactome (http://dp.univr.it/∼laudanna/LCTST/downloads/index.html). We identified the nodes involved in the shortest paths among experimentally validated miRNA targets. We considered the proteins with highest degree (hub) as the most perturbed targets in the biological processes under investigation. We then generated a network including the targets of the miRNAs as mentioned in Materials and Methods, and the differentially expressed miRNAs. Using this representation, we highlighted the relationship between miRNA-related changes and potential protein targets. Hub proteins of the network included BCL2, CDK6, MYB, CTNNB1, ZEB1, XBP1 and BAX together with 31 miRNAs connected with the seven hub proteins (Figure 10).

## DISCUSSION

MiRNAs play essential roles in the B-cell development and their deregulation was a hallmark in various lymphoma types [7, 26, 30–35]. To increase our comprehension regarding the implication of miRNAs in NHBCLs, we first compared 76 lymphoma samples belonging to five NHBCL types and identified a set of miRNAs able to discriminate among BL, DLBCL, PMBL, MCL and FL. NHBCLs, separated in two large clusters: a cluster included BL, DLBCL and PMBL cases and another cluster included FL and MCL cases. Among the 110 miRNAs able to discriminate the five lymphomas types, members of *miR-17-92* cluster and *miR-29* family represented top rank markers. These two miRNA groups showed a higher variance with respect to other miRNAs. A group of samples, mostly BL, showed the highest level of *miR-17-92* cluster and the lowest level of *miR-29* family. In other NHBCL types, the reverse correlation of the two groups of miRNAs was less pronounced. This dichotomy was observed by other authors who performed a diverse type of comparison among NHBCLs [26, 31, 34, 36, 37]. The reverse correlation of miRNAs member of *miR-17-92* cluster and *miR-29* family was previously reported as a typical pattern dependent from MYC in NHBCLs [26]. According to this consideration, we could conclude that a *MYC* signature deeply affects the miRNA profiles of NHBCLs.

We then searched for a confirmation of the miRNA signature observed in NHBCL tissues by studying BL and MCL cell lines. BL is a typical highly proliferating and malignant MYC-driven lymphoma, while MCL is a CCND1-positive lymphoma with a heterogeneous clinical behaviour. A specific difference between MCL cell lines MAVER-1 and GRANTA-519 is the presence of a *MYC* translocation in MAVER-1 but not in GRANTA-519 [38]. BL in known to share a gene expression program with normal GC B-cells [39] and accordingly the cell lines showed a miRNA profile more similar to that of GC B-cells compared to other normal B-cells. The main differences regarded the *miR-17-92* cluster, which was highly expressed in BL cell lines and in the MCL cell line MAVER-1 but not in GRANTA-519. Other differences between the six BL cell lines and GC B-cells regarded the upregulation of *miR-126*, miR-126* and of *miR-99b* and the downregulation of *miR-150*, *miR-221*/*222* family (paralogues), *miR-223* and *miR-224*. These same miRNAs were heterogeneously expressed in the GC B-cell samples, suggesting that BL might derive from a specific subpopulation of GC B-cells with proliferating potential.

The two MCL cell lines showed miRNA profiles significantly different from those of *naive* B-cells, which are supposedly their cell of origin. So, it is possible that *miR-150* downregulation (and the cooperative effect resulting of the upregulation of its target genes) is an intrinsic feature to the pathogenesis of MCL cells and might be related to the presence of activation of the B-cell receptor pathway in analogy to GC B-cells [40, 41].

The strong downregulation of *miR-221*/*222* family, *miR-223* and *miR-224* in all lymphoma cell lines compared to all normal B-cells removes a mechanism of buffering and unlocks the expression of their gene targets. In contrast, these miRNAs were found upregulated in many solid tumors in multiple studies. *MiR-221*/*miR-222* control cell migration, proliferation and apoptosis in different contexts. *MiR-221*/*miR-222* target KIT in cord blood progenitors and their appropriate level is essential for a correct hematopoietic development [8, 42]. *Mir-223* is under control of NOTCH and NF-kB signaling [43]. *Mir-223* was found downregulated in various lymphoma types and the downregulation associated with worst prognosis [43, 44]. *MiR-224* was supposed to be oncogenic in consequence of its control of the Wnt pathway through Frizzled-5 [45]. Low expression of *miR-224* predicted poor clinical outcome in DLBCL [46]. According to this evidence, we can consider *miR-221/miR-222*, *miR-223* and *miR-224* as critical mediators of the lymphomagenesis.

By comparing BL tissues with reactive LNs as normal references, we confirmed the upregulation of the *miR-17-92* cluster, *miR-196b*, *miR-219* and the downregulation of the miRNAs *miR-150*, *miR-221*, *miR-222*, *miR-29c*, *miR-155*, *miR-23a* and *miR-140*, which were found to be differentially expressed also in BL cell lines compared to GC B-cells. Once again, the miRNAs with higher discriminating power for BL were miRNAs members of the *miR-17-92* cluster. The recurrent involvement of the *miR-17-92* cluster as pathological signature in lymphoma cell lines and tissues suggests that the key factor in determining the miRNA signatures could be linked to *MYC* action [18, 22, 25, 47, 48]. According to this scenario, the crosstalk between MYC and the MYC-regulated miRNAs has a central role in the maintenance of the transformed state.

To test the hypothesis that miRNAs expression levels found in NHBCLs are under MYC influence, we determined the fraction of MYC^*+*^ cells in NHBCL tissues by a software-based approach and correlated the cell counts with the level of miRNAs known to be upregulated and downregulated by MYC. This quantitative IHC approach is original in the study of correlation between MYC and miRNAs expression. A higher fraction of MYC^+^ cells was detected in BL than in DLBCL, PMBL, MCL, FL and LN, from highest to the lowest. Our data highlighted an evident correlation among MYC^*+*^ cell counts and MYC-related miRNAs level in all NHBCLs types. A similar approach based on MYC mRNA and miRNA levels correlation was previously applied in neuroblastoma [25]. In another study, the MYC-related miRNAs were extracted from profiles by comparing the miRNA differentially expressed between BL and MYC-independent NHBCLs [26]. No correlation between MYC mRNA level and the expression of miRNAs members of the *miR-17-92* cluster was found in BL, probably as a consequence of the presence of saturating MYC (i.e. there’s no dynamic range being MYC always very highly expressed) [12].

In our analysis, the more clearcut evidence of correlation between MYC and miRNAs levels arose among the DLBCLs. In fact, in this lymphoma type MYC^+^ cell counts ranged from 2% to 82%, allowing to test the miRNA signature on a wide dynamic range. DLBCL showed a clear correlation between MYC-related miRNAs expression and MYC^+^ cell counts. MiRNA profile of DLBCL cases with high number of MYC^*+*^ cells clustered close to BL cases. A similar trend was observed also in MCL, FL and reactive LNs. On the whole, 30.7% of MCL cases showed more than 20% of MYC^+^ cells and a correlation with the MYC-related miRNA signature that parallel that one of DLBCL. We did not find *MYC* translocation in MCL. Instead, we found *MYC* gain in three MCL cases with higher MYC^+^ cell counts. These data are concordant with the reported relevant incidence of *MYC* abnormalities in MCL (36% of cases with gains, amplification or rarely translocations) [15].

We identified a novel group of miRNAs whose expression was correlated with that of MYC. These correlations are not yet supported by experimental demonstration. Nevertheless, there is evidence supporting a functional connection between some new MYC-related miRNAs and MYC. *MiR-206* was shown to target *MAP3K13* and as a consequence to determine MYC destabilization [49]. A possible link between *miR-219* and MYC was reported. In medulloblastoma, *miR-219* downregulated CD164 and in consequence MYC was degraded [50]. *MiR-130b* was reported to promote *MYC* expression through the YAZ/TAZ pathway [51]. The restoration of *miR-340* in breast cancer cell lines lacking this miRNA inhibited cell growth by the activation of the Wnt pathway and the MYC expression [52].

In BL, high level of MYC and founder alterations induce a field defect characterized by the aberrant activation of PI3K signaling pathway and other changes mediated by the non-canonical binding of MYC to other proteins and genetic loci [29, 53, 54]. A constitutive and disregulated early expression of MYC and the synergism between MYC and the MYC-related miRNAs can overcome the transcriptional program mediated by BCL6. In fact, BCL6 prevents MYC expression in the B-cells of the dark zone of the GC and adapts the cellular functions to the growth conditions of the lymphoma cells [22]. According to this description, we can hypothesize that MYC overexpression observed in lymphomas has a predominantly quantitative rather than qualitative effects on the related miRNA circuitry and other cellular process. On the other side, in consequence of the wide spectrum of possible targets that is typical of most of miRNAs, a stronger miRNA signature under MYC control might affect signaling pathways and cellular functions responsible of the retention of the pathological features and support the growth of the transformed cells.

By network analysis we found that BCL2, CDK6, MYB, CTNNB1, ZEB1, XBP1 and BAX are hub protein of the network under the control of deregulated miRNAs in BL. All these proteins are experimentally validated potential direct targets of MYC-related miRNAs and play important roles in B-cell development. The 7 proteins were not altogether significantly modulated following *miR-17-92* cluster overexpression in oncogenic models [22, 48, 55–58]. We guess that all miRNAs differentially expressed in BL are implicated in the fine tuning of their target genes in support of cell growth and that the 7 proteins are central to this regulatory network. The finding of BCL2 in a MYC-related miRNAs targets network associated to BL, lymphoma in which BCL2 protein expression is absent, should not surprise. The GC reaction requires a transient biphasic MYC protein expression in B-cells: early, during their early commitment, and later for reentry into the GC dark zone [53]. In contrast, BCL2 expression is not required. In fact, BCL2 mRNA and protein are scarcely present in the GC B-cells and miRNA could be implicated in the repression of BCL2 mRNA. The long list of miRNAs that were experimentally demonstrated to target BCL2 includes many MY*C*-related miRNAs as *miR-17-5p*, *miR-18a*, *miR-20a*, *miR-29* family, *let-7* family, *miR-34a*, *miR-34b*, *miR-125b* (see miRTarBase at http://mirtarbase.mbc.nctu.edu.tw/). It remains to be determined if and which miRNAs contribute to silencing of BCL2 in follicular B-cells.

In conclusion, we identified miRNA signatures across the whole spectrum of NHBCLs as well as in BL and MCL cell lines. These comparisons lead to the recognition of known and new miRNA clusters correlated with MYC^+^ cell counts. By network analysis, we found that BCL2, CDK6, MYB, CTNNB1, ZEB1, XBP1 and BAX are hub proteins under the control of deregulated miRNAs in BL. Accordingly, we can hypothesize that the 7 hub proteins might constitute an additional piece of the regulatory circuitry operated by MYC.

## MATERIALS AND METHODS

### Samples

Frozen tissues of 76 NHBCLs and 11 reactive LN were examined. NHBCL samples comprised 12 Burkitt’s lymphoma, 13 diffuse large B-cell lymphoma, 8 primary mediastinal B-cell lymphoma, 17 mantle cell lymphoma and 26 follicular lymphomas (Supplementary Table 3). Four DLBCL cases were classified as GCB DLBCL and 9 as non-GC DLBCL according to the IHC expression of CD10, BCL6 and MUM1, as proposed by Hans *et al.* 2004 [59]. Samples were obtained from patients accessing care in the Hematology section of the University of Verona Hospital compound between 1992 and 2005. An expert pathologist reviewed NHBCLs diagnoses. Biopsy and blood samples were obtained after informed consent approved by Ethical Committee of Verona University Hospital (N. 1828, May 12, 2010 “Institution of cell and tissue collection for biomedical research in Onco-Hematology”). Six BL cell lines Ramos, Raji, Daudi, Laukes, Namalwa and LY91, and two MCL cell lines MAVER-1 and GRANTA-519 (here named GRANTA) were studied.

### Control cells

Two CD34^+^ cells samples were from the bone marrow of two healthy donors. Twenty-six CD19^+^ normal B-cell samples were studied. Four CD19^+^ B-cell samples were from blood of four healthy donors. Twenty-two CD19^+^ normal B-cell samples were prepared from nonpathological tonsils after surgical removal at the National Institute for Cancer Research of Genova. The materials used have been collected under Program 1-IC01 protocol IC/01/LLC/001 13/12/2001, renewed 28/11/2013. The protocols, which considered the collection of the informed consent of the patient, were approved by the local ethics committee of the IRCCS Azienda Ospedaliera Integrata San Martino – IST Istituto Nazionale per la Ricerca sul Cancro (Genova).

### Cell separation

CD34^+^ cells samples were isolated by anti-CD34 antibody-conjugated magnetic beads and magnetic sorting. CD19^+^ cells were isolated from the peripheral blood of healthy donors by positive selection with anti-CD19 antibody-conjugated magnetic beads and magnetic sorting. Normal follicular B-cell isolation was performed as reported by Dono *et al.* [60]. Purity of B lymphocytes was at least 95%, according to cytofluorimetric analysis.

### Nucleic acids

Five mm sections of NHBCL and LN frozen tissues were cut at the cryostat. Tumor cellularity of NHBCLs was higher than 70%. Total RNA was isolated from frozen sections, sorted cells and cell lines using TRIZOL reagent (Invitrogen) according to manufacturer’s protocol. RNA concentration and integrity was determined respectively by spectrophotometer and agarose gel electrophoresis.

### Quantitative RT-PCR (qRT-PCR)

For each sample and for each miRNA, 5 ng of total RNA was converted to cDNA by TaqMan MicroRNA Reverse Transcription kit (Applied Biosystems) with the miRNA specific primer contained in the TaqMan MicroRNA assays (Applied Biosystems). MiRNA expression was evaluated in 10 μL total volume in the presence of 1× TaqMan Universal Master Mix (Applied Biosystems). Taqman assays (Applied Biosystems) used for miRNA expression analysis were: 4373169 for *let-7a*, 4395342 for miR-9*, 4373153 for *miR-10a*, 4373261 for *miR-20b*, 4373090 for *miR-21*, 4395223 for *miR-29a,* 4373127 for *miR-150*, 4395459 *for miR-155*, 4395387 for *miR-222*, 4380911 for *RNU44* and 4373384 for *U47*. QPCR analysis was performed in duplicate on 7900HT SDS instrument (Applied Biosystems) and the average cycle threshold value was used for subsequent calculations. Expression differences among samples were determined by the comparative method according to User Bulletin #2 (Applied Biosystems) using the average level of noncoding RNA *RNU44* and *U47* as reference.

### IHC

MYC^*+*^ cells were quantified in 4 μm tissue sections using the rabbit monoclonal antibody anti-MYC Y69 diluted 1:300 (Epitomics, UK). Immunostaining was performed using a BondMax autostainer (Leica Biosystems). High resolution images of sections counterstained with haematoxylin were captured by Aperio slide scanner (Leica Biosystems). Positive cell count was performed using the cellSense Dimension software (Olympus). The fraction of MYC^+^ cells having a nuclear signal above the background signal in each lymphoma and LN sample was the ratio between the number of MYC^+^ nuclei and 10.000 haematoxylin-stained nuclei. The background signal was assessed on slides of reactive LNs showing a low frequency nuclear stain. The threshold was set just under the level that allowed considering as negative the 95% of cells and at least 1^+^ the remaining positive cells, according to the evaluation of an expert pathologist.

### FISH

FISH was carried out using dual color IgH-MYC and IgH-BCL2 probes (Vysis-Abbott, Olympus, Rome, Italy) on serial tissue sections. Briefly, 3 μm sections were cut from formalin-fixed paraffin-embedded tissue blocks and mounted on positively charged slides. The slides were dried for one hour at 60° C then deparaffinized, rehydrated, and fixed in methanol/acetic acid 3:1 for 5 min. Pretreatment was performed at 85° C for 30 min with 0,1 citrate buffer (pH 6) solution followed by pepsin (4 mg/ml in 0.9% NaCl, pH 1.5) treatment for 8 min at 37° C. After washing and dehydration, 10 μl probe was applied on selected area and sealed with rubber cement. Denaturation was performed by incubating the slides at 80° C for 10 min in a humidified atmosphere (Thermobrite System) followed by hybridization overnight at 37° C. The rubber cement and the cover slip were removed and the slides were washed in 2× SSC/0.3% NP40 for 15 min at room temperature and then at 72° C for 2 min. Next, the tissue sections were counterstained with DAPI antifade (Prolong Gold Antifade Reagent Life Technologies) and examined under an ×60 to ×100 oil immersion objective using an Olympus BX61 fluorescence microscope equipped with filters that visualize the different wavelengths of the fluorescent probe. Scoring was performed by one experienced pathologist (MB) and one experienced biologists (GM). At least 100 neoplastic nonoverlapping nuclei were included in the scoring.

### Microarrays

MicroRNA labeling and hybridization were performed using 5 mgmicrograms total RNA, as described [52]. We used a multi-species microarray platform containing 2284 probes, 1256 for human and 1028 for mouse targets, respectively. A total of 353 human mature or pre-miR were detectable by the microarray. Each human target was matched by at least two probes, with an average of 4.3 probes for each target. Hybridization signals were detected with Streptavidin-Alexa647 conjugate and scanned images (Axon 4000B) were quantified using the Genepix 6.0 software (Axon Instruments). To minimize the possible batch effect on miRNAs expression, samples of the same category were randomized through different batches.

### Data analysis

Expression data from microarrays were normalized and transformed using the *vsn* package for R. The spots were subsequently classified based on their target sequence regardless of the original designation. The expression measures for probes matching the same miR sequence were summarized using the *medpolish* algorithm, in order to obtain a unique expression figure for each target. Clustering analysis was performed using the *hclust* function and the inverse Pearson correlation as a distance metric, for both genes and arrays. All the clusters were visualized using the Java TreeView software (http://jtreeview.sourceforge.net). To select differentially expressed genes, we performed either anova or *t*-tests. To take into account multiple hypothesis testing, the FDR (false discovery rate) was calculated using the *q-*value package for R. All the calculations were performed using the R statistical software (http://www.r-project.org). Microarray expression data are available on the ArrayExpress at https://www.ebi.ac.uk/arrayexpress/experiments/E-MTAB-6035.

### Network analysis

Experimentally validated target genes (only target genes marked as “strong evidence”) of differentially expressed miRNAs were retrieved from MirTarBase 4.5 database [61]. Among the predicted targets, only those included in the GO category “lymphocyte differentiation” (GO:0030098) were kept. Using the filtered targets and a database of protein interactions (http://dp.univr.it/∼laudanna/LCTST/downloads/index.html) and comprising 14642 proteins and 270062 edges, we generated a network that included the shortest paths joining the primary targets. Shortest paths were calculated with PeSca application 3.0 version implemented in Cytoscape 3.0 [62, 63].

## SUPPLEMENTARY MATERIALS FIGURE AND TABLES


