# Supplementary material for: MYC-related microRNAs signatures in non-Hodgkin B-cell lymphomas and their relationships with core cellular pathways
Source: Oncotarget. 2018 Jul 3;9(51):29753–71. doi: 10.18632/oncotarget.25707 (PMC6049865; doi:10.18632/oncotarget.25707)
Supplement: Supplementary file 1 [file oncotarget-09-29753-s001.pdf]

MYC-related microRNAs signatures in non-Hodgkin B-cell lymphomas and their relationships with core cellular pathways

SUPPLEMENTARY MATERIALS

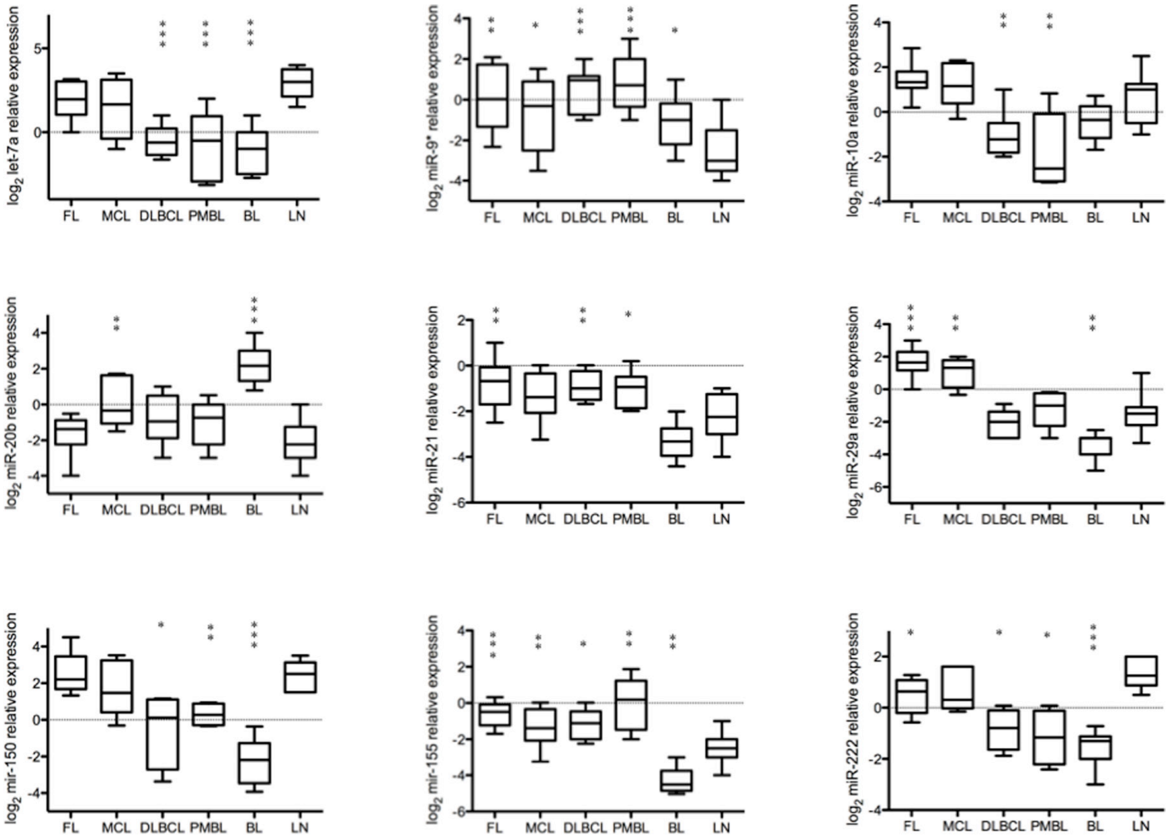

Supplementary Figure 1: Validation of miRNAs expression in NHBCLs and LNs by quantitative RT-PCR. Expression analysis of *let-7a, miR-9*, miR-10a, miR-20b, miR-21, miR-29a, miR-150, miR-155* and *miR-222* was assessed in FL, MCL, DLBCL, PMBL BL and LN samples. Box plot: line, average; whiskers, 10-90 percentile. The data are log2 relative expression levels normalized with noncoding RNAs *RNU44* and *U47*. Statistical analysis was performed by *t*-test. Asterisks at the top of the graphs identify a category significantly differentially expressed compared to LN: $^{*}P < 0.05$; $^{**}P < 0.01$; $^{***}P < 0.001$.

Supplementary Table 1: List of miRNAs correlated inversely with MYC+ cell counts in NHBCLs and LNs*

rho	miRNA	*Q* value	*P* value	MYC correlation
-0.71	*miR-29c*	1.06E-06	5.08E-09	nn
-0.69	*miR-29b*	2.50E-06	2.40E-08	nn
-0.65	*miR-150*	6.49E-06	9.34E-08	nn
-0.64	*miR-29b-1-3p*	6.50E-06	1.38E-07	MYC-
-0.67	*miR-29a*	6.68E-06	2.33E-07	MYC-
-0.64	*miR-342*	1.17E-05	6.54E-07	nn
-0.53	*let-7c*	1.24E-05	7.75E-07	MYC-
-0.60	*miR-26a*	1.93E-05	1.30E-06	nn
-0.49	*let-7d*	3.40E-05	2.61E-06	MYC-
-0.64	*miR-29b-1-3p*	3.97E-05	3.23E-06	nn
-0.59	*miR-150*	4.17E-05	3.60E-06	nn
-0.52	*let-7a-2-5p*	4.35E-05	4.09E-06	MYC-
-0.47	*let-7a-3-5p*	9.32E-05	9.39E-06	MYC-
-0.53	*miR-26a*	4.30E-04	5.36E-05	MYC-
-0.42	*miR-10a*	5.32E-04	7.65E-05	nn
-0.47	*miR-125a*	5.99E-04	9.19E-05	nn
-0.47	*miR-370*	6.47E-04	1.03E-04	nn
-0.50	*mir-140*	8.57E-04	1.60E-04	nn
-0.49	*miR-26b*	9.22E-04	1.77E-04	MYC-
-0.47	*miR-361*	1.74E-03	3.67E-04	nn
-0.42	*miR-142-5p*	1.74E-03	3.79E-04	nn
-0.40	*miR-10b*	1.74E-03	3.92E-04	nn
-0.36	*mir-1-2-3p*	1.74E-03	3.85E-04	nn
-0.44	*let-7g*	1.91E-03	4.40E-04	MYC-
-0.56	*miR-146b*	1.96E-03	4.62E-04	nn
-0.33	*miR-196b*	2.13E-03	5.11E-04	nn
-0.44	*mir-10b*	2.27E-03	5.55E-04	nn
-0.48	*miR-374*	2.53E-03	6.32E-04	nn
-0.49	*miR-26a*	4.58E-03	1.43E-03	MYC-
-0.28	*miR-373*	4.58E-03	1.45E-03	nn
-0.29	*miR-29b-1-3p*	5.34E-03	1.82E-03	nn
-0.32	*mir-153-1-3p*	5.43E-03	1.87E-03	nn
-0.28	*miR-520d*	5.77E-03	2.05E-03	nn
-0.45	*miR-125b*	5.87E-03	2.11E-03	nn
-0.48	*miR-30d*	6.14E-03	2.24E-03	nn
-0.41	*miR-100*	6.92E-03	2.59E-03	nn
-0.42	*miR-27a*	7.24E-03	2.75E-03	nn
-0.27	*miR-216*	7.83E-03	3.08E-03	nn
-0.36	*miR-140*	8.67E-03	3.45E-03	nn
-0.52	*miR-146a*	9.32E-03	3.80E-03	nn
-0.34	*miR-200c*	9.90E-03	4.08E-03	nn
-0.27	*mir-1-2-3p*	1.01E-02	4.26E-03	nn
-0.33	*miR-126**	1.18E-02	5.16E-03	nn
-0.29	*miR-302b**	1.19E-02	5.23E-03	nn

-0.31	*miR-196a*	1.68E-02	7.81E-03	nn
-0.28	*miR-184*	1.68E-02	7.81E-03	nn
-0.22	*miR-432*	1.68E-02	7.81E-03	nn
-0.26	*let-7e*	1.68E-02	7.91E-03	nn
-0.33	*miR-34a*	1.93E-02	9.36E-03	nn
-0.35	*miR-125b*	1.94E-02	9.48E-03	nn
-0.45	*miR-222*	2.06E-02	1.04E-02	nn
-0.34	*miR-199a*	2.06E-02	1.04E-02	nn
-0.31	*miR-100*	2.09E-02	1.06E-02	nn
-0.21	*mir-144*	2.18E-02	1.12E-02	nn
-0.32	*miR-196a*	2.40E-02	1.27E-02	nn
-0.53	*miR-221*	3.21E-02	1.80E-02	nn
-0.34	*miR-99a*	3.59E-02	2.10E-02	nn
-0.23	*miR-98*	3.73E-02	2.25E-02	nn
-0.17	*miR-502*	3.73E-02	2.25E-02	nn
-0.14	*miR-143*	3.73E-02	2.25E-02	nn
-0.29	*miR-147*	3.83E-02	2.33E-02	nn
-0.34	*let-7f*	3.93E-02	2.47E-02	nn
-0.40	*miR-30b*	4.77E-02	3.16E-02	nn
-0.35	*miR-301*	4.92E-02	3.30E-02	nn

*rho, Sperman's rank correlation coefficient; MYC-, miRNA known as downregulated by MYC; nn, MYC correlation not previously known. A single miRNA can have more that one probe.

Supplementary Table 2: List of miRNAs correlated directly with MYC⁺ cells counts in NHBCLs and LNs*

rho	miRNA	*Q* value	*P* value	MYC correlation
0.63	*miR-106a*	6.50E-06	1.56E-07	MYC+
0.60	*mir-206*	6.68E-06	2.56E-07	nn
0.65	*miR-17-5p*	6.68E-06	2.39E-07	MYC+
0.58	*miR-219*	1.17E-05	6.04E-07	nn
0.61	*miR-499*	1.17E-05	6.77E-07	nn
0.63	*miR-20a*	1.17E-05	6.04E-07	MYC+
0.44	*miR-130b*	2.40E-05	1.73E-06	nn
0.62	*miR-20b*	4.35E-05	4.17E-06	nn
0.56	*mir-219*	1.71E-04	1.81E-05	nn
0.49	*miR-324-5p*	1.90E-04	2.10E-05	nn
0.48	*miR-328*	3.38E-04	3.90E-05	nn
0.46	*miR-330*	4.30E-04	5.27E-05	nn
0.57	*miR-106a*	4.39E-04	5.69E-05	nn
0.46	*miR-485-3p*	4.48E-04	6.01E-05	nn
0.56	*mir-431*	4.48E-04	6.24E-05	nn
0.48	*miR-18a*	5.54E-04	8.24E-05	MYC+
0.38	*miR-192*	7.26E-04	1.18E-04	nn
0.40	*mir-129-1*	8.05E-04	1.39E-04	nn
0.56	*miR-106b*	8.05E-04	1.39E-04	MYC+
0.47	*miR-212*	8.27E-04	1.47E-04	nn
0.26	*miR-136*	8.57E-04	1.58E-04	nn
0.50	*miR-212*	9.22E-04	1.81E-04	nn
0.44	*mir-422a*	1.00E-03	2.01E-04	nn
0.42	*miR-19b*	1.63E-03	3.37E-04	MYC+
0.49	*miR-19b*	2.60E-03	6.74E-04	MYC+
0.52	*mir-339*	2.60E-03	6.74E-04	nn
0.38	*miR-338*	2.64E-03	6.96E-04	nn
0.27	*miR-205*	2.89E-03	7.91E-04	nn
0.45	*mir-196b*	2.89E-03	7.78E-04	nn
0.38	*miR-326*	3.13E-03	8.70E-04	nn
0.32	*miR-324-5p*	3.59E-03	1.02E-03	nn
0.41	*mir-335*	3.59E-03	1.03E-03	nn
0.41	*mir-130b*	3.76E-03	1.10E-03	nn
0.37	*miR-324-5p*	4.32E-03	1.28E-03	nn
0.43	*miR-214*	4.45E-03	1.34E-03	nn
0.33	*mir-320*	4.51E-03	1.39E-03	nn
0.30	*miR-132*	4.72E-03	1.52E-03	nn
0.36	*mir-453*	5.25E-03	1.71E-03	nn
0.35	*miR-128a*	5.34E-03	1.82E-03	nn
0.35	*mir-181a-2*	5.34E-03	1.82E-03	nn
0.36	*mir-99b-3p*	5.60E-03	1.96E-03	nn
0.41	*miR-7*	6.81E-03	2.52E-03	nn
0.37	*mir-107-5p*	7.36E-03	2.83E-03	nn
0.36	*miR-218*	7.38E-03	2.87E-03	nn

0.44	*miR-194*	8.68E-03	3.50E-03	nn
0.37	*miR-483*	1.00E-02	4.19E-03	nn
0.41	*miR-497*	1.10E-02	4.69E-03	nn
0.41	*miR-202**	1.18E-02	5.12E-03	nn
0.35	*mir-211*	1.42E-02	6.32E-03	nn
0.23	*mir-16-2*	1.44E-02	6.49E-03	nn
0.24	*miR-378**	1.73E-02	8.23E-03	nn
0.36	*mir-133b*	1.90E-02	9.13E-03	nn
0.33	*miR-9*	1.94E-02	9.61E-03	MYC+
0.32	*miR-346*	2.21E-02	1.15E-02	nn
0.29	*miR-323*	2.36E-02	1.24E-02	nn
0.34	*mir-184*	2.54E-02	1.36E-02	nn
0.34	*mir-152*	2.54E-02	1.36E-02	nn
0.26	*mir-524**	2.58E-02	1.40E-02	nn
0.19	*miR-96*	2.62E-02	1.43E-02	nn
0.28	*mir-210-3p*	2.66E-02	1.47E-02	nn
0.26	*mir-129-2*	2.94E-02	1.64E-02	nn
0.26	*mir-34a*	3.26E-02	1.85E-02	MYC-
0.36	*miR-129*	3.35E-02	1.91E-02	nn
0.26	*miR-193b*	3.54E-02	2.05E-02	nn
0.32	*mir-424*	3.54E-02	2.05E-02	nn
0.38	*miR-129*	3.64E-02	2.15E-02	nn
0.13	*miR-488*	3.93E-02	2.47E-02	nn
0.28	*miR-337*	3.93E-02	2.47E-02	nn
0.35	*miR-93*	3.93E-02	2.47E-02	MYC+
0.39	*miR-498*	3.99E-02	2.53E-02	nn
0.29	*mir-22-3p*	4.05E-02	2.58E-02	MYC+
0.18	*mir-329-1-3p*	4.60E-02	2.96E-02	nn
0.30	*miR-412*	4.67E-02	3.02E-02	nn
0.11	*mir-376b-5p*	4.77E-02	3.16E-02	nn
0.18	*mir-148a*	4.77E-02	3.16E-02	MYC-
0.25	*mir-132*	4.85E-02	3.23E-02	nn
0.33	*miR-429*	4.94E-02	3.34E-02	nn
0.26	*mir-106b*	5.07E-02	3.45E-02	nn

*rho, Sperman‘s rank correlation coefficient; MYC+, miRNA known as upregulated by MYC; MYC-, miRNA known as down-regulated by MYC; nn, MYC correlation not previously known.
A single miRNA can have more that one probe.

Supplementary Table 3: Seventy-eight lymphoma cases studied for miRNA expression and their known molecular features*

Patients	Diagnosis	IHC			FISH
		BCL2	BCL6	MYC (% of positive cells)	
1	BL	na	na	53.7	nd
2	BL	na	na	90.0	nd
3	BL	na	na	92.5	nd
4	BL	na	na	93.4	nd
5	BL	na	na	67.0	nd
6	BL	na	na	95.8	nd
7	BL	na	na	na	nd
8	BL	na	na	78.3	nd
9	BL	na	na	98.3	nd
10	BL	na	na	93,3	nd
11	BL	na	na	96.3	nd
12	BL	na	na	83.0	nd
13	FL grade 1	++	+	5.3	nd
14	FL grade 1	++	+	10.5	nd
15	FL grade 1	++	+	24.4	nd
16	FL grade 1	++	+	0.6	nd
17	FL grade 1	++	++	8.9	nd
18	FL grade 1	0	+	1.1	nd
19	FL grade 1	+	+	4.5	nd
20	FL grade 2	+	+	21.9	nd
21	FL grade 2	++	++	38.5	nd
22	FL grade 2	++	+	3.3	nd
23	FL grade 2	+	+	11.1	nd
24	FL grade 2	++	+	9.8	nd
25	FL grade 2	++	+	na	nd
26	FL grade 3a	+	+	15.6	nd
27	FL grade 3a	0	+	na	nd
28	FL grade 3a	++	+	2.2	nd
29	FL grade 3a	+	+	1.2	nd
30	FL grade 3a	++	+	7.7	nd
31	FL grade 3a	++	+	9.5	nd
32	FL grade 3a	++	+	5.9	nd
33	FL grade 3a	+	+	3.8	nd
34	FL grade 3a	++	+	10.3	nd
35	FL grade 3a	++	+	19.7	nd
36	FL grade 3b	0	++	7.4	nd
37	FL grade 3b	+	+	34.0	nd
38	FL grade 3b	+	+	3.0	nd
39	FL grade 3b	+	+	2.1	nd
40	FL grade 3b	0	+	39.6	nd
41	GCB DLBCL	0	+	40.4	MYC nt, BCL2 nt

42	non GCB DLBCL	++	0	70.7	MYC nt, BCL2 nt
43	GCB DLBCL	0	+	32.7	MYC nt, BCL2 nt
44	GCB DLBCL	0	+	0.6	MYC nt, BCL2 nt
45	non GCB DLBCL	0	0	na	MYC nt, BCL2 nt
46	non GCB DLBCL	0	++	83.5	MYC nt, BCL2 nt
47	non GCB DLBCL	++	0	25.6	MYC nt, BCL2 nt
48	non GCB DLBCL	++	0	37.0	MYC nt, BCL2 nt
49	non GCB DLBCL	++	0	81.7	MYC translocated, BCL2 nt
50	non GCB DLBCL	++	0	47.5	MYC nt, BCL2 nt
51	non GCB DLBCL	+	0	25.7	MYC nt, BCL2 nt
52	non GCB DLBCL	++	+	55.7	MYC nt, BCL2 nt
53	GCB DLBCL	+	+	77.5	MYC nt, BCL2 nt
54	MCL	na	na	55.0	MYC gain
55	MCL	na	na	69.2	MYC gain
56	MCL	na	na	1.6	nd
57	MCL	na	na	na	nd
58	MCL	na	na	30.8	MYC gain
59	MCL	na	na	40.0	na
60	MCL	na	na	4.6	nd
61	MCL	na	na	2.2	nd
62	MCL	na	na	na	nd
63	MCL	na	na	2.3	nd
64	MCL	na	na	0.5	nd
65	MCL	na	na	1.8	nd
66	MCL	na	na	na	nd
67	MCL	na	na	na	nd
68	MCL	na	na	na	nd
69	MCL	na	na	22.5	nd
70	MCL	na	na	2.4	nd
71	PMBL	na	na	na	nd
72	PMBL	na	na	na	nd
73	PMBL	na	na	na	nd
74	PMBL	na	na	60.0	nd
75	PMBL	na	na	na	nd
76	PMBL	na	na	na	nd
77	PMBL	na	na	37.9	nd
78	PMBL	na	na	32.3	nd

*GCB DLBCL, germinal center B-cell-like DLBCL; non-GCB DLBCL, non germinal center B-cell-like DLBCL; GCB and non-GCB DLBCL classification was obtained according to Hans CP et al. Blood 2004; 0, not expressed; +, weak expression; ++, strong expression; na, not available; nt, non translocated; MYC %, normalized percentage of MYC+ cells; nd, not done; MYC gain, MYC copy number gain. All samples were prior to 2005.
